# Autapse-Induced Spiral Wave in Network of Neurons under Noise

**DOI:** 10.1371/journal.pone.0100849

**Published:** 2014-06-26

**Authors:** Huixin Qin, Jun Ma, Chunni Wang, Ying Wu

**Affiliations:** 1 Department of Physics, Lanzhou University of Technology, Lanzhou, China; 2 School of Aerospace, Xian Jiaotong University, Xian, China; Hong Kong Baptist University, Hong Kong

## Abstract

Autapse plays an important role in regulating the electric activity of neuron by feedbacking time-delayed current on the membrane of neuron. Autapses are considered in a local area of regular network of neurons to investigate the development of spatiotemporal pattern, and emergence of spiral wave is observed while it fails to grow up and occupy the network completely. It is found that spiral wave can be induced to occupy more area in the network under optimized noise on the network with periodical or no-flux boundary condition being used. The developed spiral wave with self-sustained property can regulate the collective behaviors of neurons as a pacemaker. To detect the collective behaviors, a statistical factor of synchronization is calculated to investigate the emergence of ordered state in the network. The network keeps ordered state when self-sustained spiral wave is formed under noise and autapse in local area of network, and it independent of the selection of periodical or no-flux boundary condition. The developed stable spiral wave could be helpful for memory due to the distinct self-sustained property.

## Introduction

Neuronal system consists of a large number of neurons, and the collective electric activities of neurons can emerge complex spatiotemporal patterns during the process of signals. It is believed that signals communication can be realized by synapse coupling, and the distribution of spatiotemporal pattern can present some useful clues to understand the information propagation between neurons [1]–[8]. In this way, some researchers show great interests in detecting the formation and transition of patterns in network of neurons [9]–[19]. It was reported that spiral wave can be found in the cortex of brain [20]–[22], and then some theoretical investigations [23], [24] have been presented to explore the potential formation mechanism of spiral wave and multi-armed spiral wave in the network of neurons, indeed, these results [24] have confirmed that intermediate channels blocking in neurons in a local area of neuronal network can induce emergence of spiral wave, and the stability of multiarmed spiral wave was also discussed. It was also confirmed that artificial defects [25] can block travelling wave to induce perfect spiral wave under appropriate coupling intensity between neurons, and breakup of spiral wave occurs with strong noise or channel noise [26], [27] being used.

Chemical and electric synapses bridge the neurons during signal transmission and the mutual connections can dominate the collective behaviors of neurons or oscillators. That is to say, an unusual kind of synapse is a specialized connection between neurons or between a neuron and a muscle, which is used for transmitting electrical signals. While an autapse is a self-synapse, which exists a connection between a neuron and itself [28]–[30]. Herrmann et al. [31] presented an experimental study to detect the functional significance that autapses offer for neural behavior, and they simulated a neural basket cell via the Hodgkin-Huxley equations and implemented an autapse which feeds back onto the soma of the neuron, interestingly, their results confirmed that neuron can become active due to the effect of autapse. Then the biophysical modeling for autapse [32] is achieved in terms of a stochastic Hodgkin-Huxley model containing such a built in delayed feedback, and the dynamics of electric activity of neurons was discussed. As a result, it is reasonable to model the effect of autapse on neuron by imposing a feedback term with time delay and feedback gain. In the case of dynamics of neuron and networks, the effect of autapse is often left out though time delay between neurons is considered in signal transmission [33], [34]. As is well known, a negative feedback is often helpful to stabilize the system while a positive feedback can enhance the oscillating of the system. For example, Ao et al. [35] numerically investigated the influence of intrinsic channel noise on the dynamical response of delay-coupling in neuronal systems. Wang et al. [36] discussed the dynamics of electrical activity and the transition of firing patterns induced by three types of autapses in Hindmarsh-Rose neuron. Indeed, Refs. [37], [38] gave a brief discussion about the potential biological function of autapse in neuron. The author of this paper ever investigated the effect of autapse on Hindmarsh-Rose neuron, and it was found that appropriate electric autapse can wake up quiescent neurons in ring network [39]. However, the effect of autapses on collective electric behaviors of neurons keeps open in the two-dimensional arrar network. In the case of ring network, stable and continuous pulses can regulate the collective behaviors of neurons? Is there any new ‘pacemaker’ in a two-dimensional array network induced by the distribution of electric autapses thus the collective behaviors of neurons can be regulated? If possible, how many neurons can be regulated by a target wave or spiral wave in the two-dimensional array network with electric autapses being considered? What is the difference if noise is also considered? It was confirmed that optimized noise on network can be active to develop a spiral wave [40] due to coherence resonance [41], [42]. More often, target wave can also be induced in the media due to heterogeneity [43] and the emitting wave can be used to suppress the spiral wave and turbulence of the media [44], for example, the tip dynamics of spiral wave is changed by the fractal heterogeneity [43], and the heterogeneity by a rotating electric field [45] can emit ordered wave to regulate the behavior of excitable media.

Noise plays important role in regulating electric activities of neurons though breakup of spiral wave could be induced by noise beyond certain intensity. In this paper, a regular network of Hindmarsh-Rose neuron is designed in a two-dimensional array, some autapses are introduced into the neurons in a local area and noise is also considered on the whole network. A statistical function is defined to detect the transition of spiral wave under noise and autapses in a local area of the network. Breakup and development of spiral wave in the network induced by few autapses, and the effect of noise will be investigated, respectively. To discern the effect of boundary condition, no-flux boundary and periodical boundary condition is considered, respectively.

## Model and Scheme

Hindmarsh-Rose(HR) neuron model is regarded as a simplified neuron model to describe the main properties of electric activities in neurons. A two-dimensional network of HR neurons with regular [46], [47] or small-world connection type [48] can be used to study the pattern formation and selection of neurons. Then the network dynamics of HR neurons with autapses in local area under noise is described by
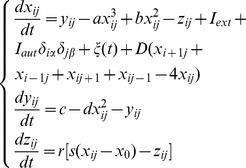
(1)

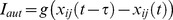
(2)where *x_ij_* represents the membrane potential of neuron in node (*i, j*), *y_ij_*_,_ *z_ij_* denotes the recovery variable and slow adaption current in node (*i, j*), respectively. *D* is the intensity of coupling between adjacent neurons, *I*_ext_ is the external forcing current on each neuron. *I_aut_* is the forcing current generated from a electric autapse, *g, τ* is the gain and time delay, respectively. Autapse current *I_aut_* ≠0 in a local area, and *α*, *β* are integers, for example, *α = *100, 101, 102, *β = *100, 101, 102, it means that autapses are imposed on the neurons in a local area (3×3 nodes) as 100≤*i, j* ≤102, thus autapse currents are considered in these neurons. *ξ*(*t*) is Gaussian white noise on each node, the statistical relation is described by <*ξ*(*t*)> = 0, <*ξ*(*t*)*ξ*(*t* ′)> = 2*D*_0_ *δ*(*t*−*t* ′), *D*_0_ is the noise intensity. For a single neuron without autapse effect being considered, it can emerge chaotic state at *a* = 1.0, *b* = 3.0, *c* = 1.0, *d* = 5.0, *s* = 4.0, *r* = 0.006, *x*_0_ = −1.56 by increasing the forcing current *I*_ext_ beyond certain threshold [46]. According to Eq. (2), negative feedback on membrane potential occurs for positive *g* values thus the neuron tends to be stable. While positive feedback on membrane potential emerges for negative *g* values so that the neuron can become active. In this paper, negative values will be selected for gain *g* to detect the development of spiral wave in the network induced by noise and a fraction of electric autapses in a local area of the network.

It is necessary to give some clarifications before further investigation in the following sections. For a single HR neuron without autapse, it is found that the neuron begins to emerge spiking at *I*_ext_>1.1, then bursting at *I*_ext_>1.8. To cares about the autapse effect, the external forcing current is selected as *I*_ext_ = 1.0 for a quiescent state without autapse. To detect the transition of collective behaviors of neurons in the network, a statistical factor of synchronization in a two-dimensional space is defined according to mean filed theory, and the factor of synchronization *R* is described by [18], [27], [47].
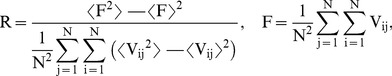
(3)Where *N* ^2^ is the total number of neurons of the network, the symbol <*> represents an average over time, the *V_ij_* is the observable variable in node (*i, j*) and it is replaced by the membrane potential *x_ij_* in the following numerical studies. It predicates a perfect synchronization for *R*∼1, while it means non-perfect synchronization for *R*∼0. In realistic neuronal systems, the distribution of autapses connected to neurons in a local area of network can be reliable and reasonable. In fact, the media becomes heterogeneous if autapses are considered on a few neurons in a local area of the network, thus target wave can be induced to occupy the network. Breakup of target waves occur under noise, thus spiral wave could be developed after collision between broken target waves. Extensive numerical results show that spiral wave can be observable easily when stronger gain *g* is used and the density of electric autapses (more autapses are included) is bigger. The potential cause is that powerful target wave occurs and spiral wave is induced via complex collision between broken target waves. For simplicity, *g* = −1.5, *τ* = 30 are used in the following numerical studies, the development of spiral wave is investigated by changing the coupling intensity *D*, noise intensity *D*_0_, and this case is discussed under no-flux and periodical boundary condition, respectively.

## Numerical Results and Discussion

In the numerical studies, the Euler forward difference algorithm is used with time step *h* = 0.01, the initial values are selected as (3.0, 0.3, 0.1), all the neurons keep quiescent states, the transient period for calculation is about 16000 time units. The network size is 200×200. Autapses are imposed on neurons in a local area with 5×5 nodes (96≤*i, j*≤100). In mathematical models and computer simulations, periodic boundary conditions (PBC) are a set of boundary conditions that are often used to simulate a large system by modeling a small part that is far from its edge. The realistic neuronal system consists of a large number of neurons, the border effect from neurons outside but close to the system border should be considered, as a result, periodical boundary condition is often appreciated. Sometimes, no-flux boundary condition is also discussed for the case that the system keeps independent from cells outside the system (or an isolated system). In the following sections, A no-flux, and periodical boundary condition is used in the study of pattern formation and selection, respectively. It confirms that the development of spiral wave is much dependent on the distribution of electric autapses in the network, or the results are much independent of the selection of boundary condition. For a clear illustration, readers can refer to the two short movies in the supporting information, in the case of appropriate noise, the development of spiral wave induced by autapses can be observed under periodical and/or no-flux boundary conditions. In this way, it can give some clues to understand how the collective behaviors of neurons in network can be regulated by a continuous ‘pacemaker’ like spiral wave induced by the electric autapses.

### Subsection A: Noise on the formation of spiral wave

**Figure 1:**
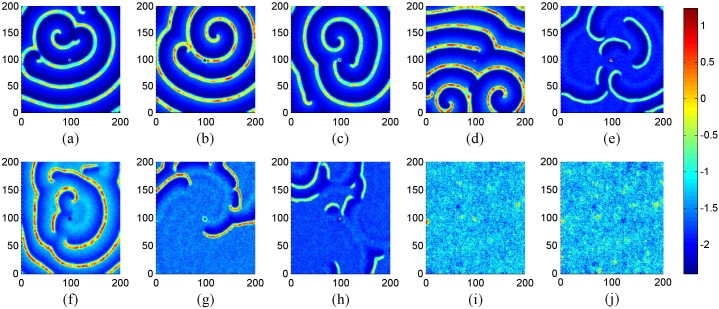
The developed states at *t* = 16000 time units in the network of neurons under different intensities of noise. For noise intensity (a) *D*_0_ = 0.005, (b) *D*_0_ = 0.01, (c) *D*_0_ = 0.02, (d) *D*_0_ = 0.03, (e) *D*_0_ = 0.04, (f) *D*_0_ = 0.05, (*g*) *D*_0_ = 0. 08, (h) *D*_0_ = 0.1, (*i*) *D*_0_ = 0.5, (j) *D*_0_ = 0.8. Where *g* = −1.5, *τ* = 30, *I_ext_* = 1.0, *D* = 1.0 and no-flux boundary condition is used.

In this subsection, the coupling intensity is fixed at *D* = 1, different intensities of noise are considered on the network. The gain intensity and time delay in autapse are selected as *g* = −1.5, *τ* = 30, and *I_ext_* = 1.0. Firstly, no-flux boundary condition is used for the network in numerical studies. As shown in Fig. 1, the developed states at *t* = 16000 time units are illustrated under different intensities of noise.

**Figure 2:**
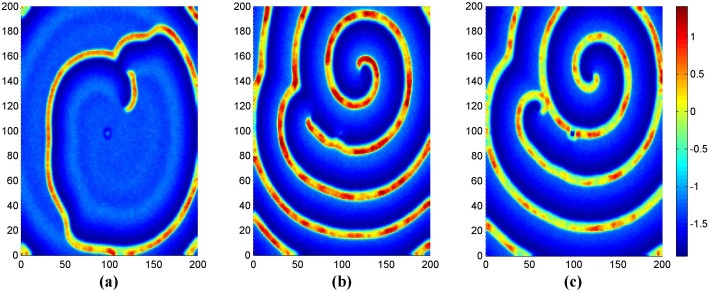
The development of spiral wave in the network. For (a) *t* = 2000, (b) *t* = 4000, (c) *t* = 16000 time units. Where *g* = −1.5, *τ* = 30, *I_ext_* = 1.0, *D* = 1.0, *D*_0_ = 0.01 and no-flux boundary condition is used.

The results in Fig. 1 show that spiral wave can be induced in the network under noise, breakup of spiral wave occurs and disordered state emerge with increasing the intensity of noise on the network. The developed spiral wave in Fig. 1(b) is much perfect than other snapshots, it means that the intensity of noise *D*_0_ = 0.01 could be the most intermediate noise to develop a stable spiral wave. Then the development of spiral wave under *D*_0_ = 0.01 is monitored, and snapshots under different time units are illustrated in Fig. 2.

**Figure 3:**
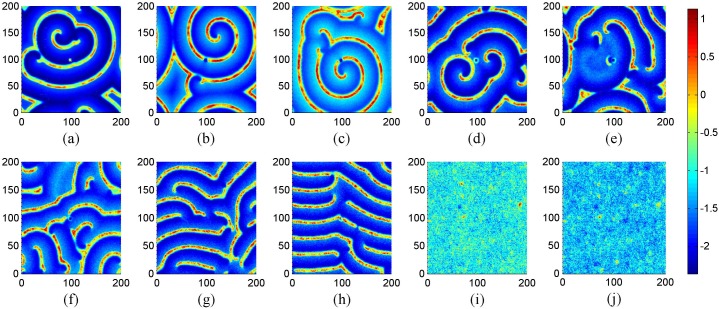
The developed states at *t* = 16000 time units in the network of neurons under different intensities of noise. For noise intensity (a) *D*_0_ = 0.005, (b) *D*_0_ = 0.01, (c) *D*_0_ = 0.02, (d) *D*_0_ = 0.03, (e) *D*_0_ = 0.04, (f) *D*_0_ = 0.05, (*g*) *D*_0_ = 0. 08, (h) *D*_0_ = 0.1, (*i*) *D*_0_ = 0.5, (*j*) *D*_0_ = 0.8. Where *g* = −1.5, *τ* = 30, *I_ext_* = 1.0, *D* = 1.0 and periodical boundary condition is used.

The results in Fig. 2 confirm that stable spiral wave emerges with a transient period about 2000 time units, the potential mechanism is that autapses in a local area are critical to induce target wave, and the target wave begins to break up under noise, then spiral wave is formed under optimized noise. Surely, it is interesting to investigate this case under periodical boundary condition, and the results are plotted in Fig. 3.

**Figure 4:**
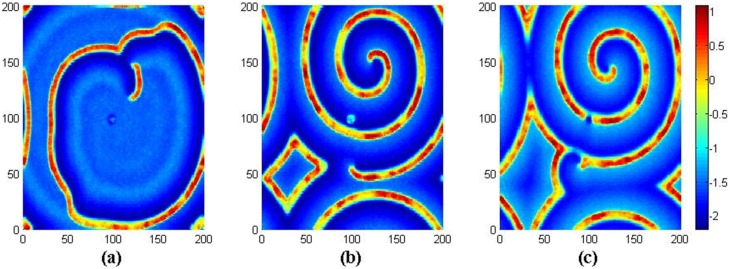
The development of spiral wave in the network. For (a) *t* = 2000, (b) *t* = 4000, (c) *t* = 16000 time units. Where *g* = −1.5, *τ* = 30, *I_ext_* = 1.0, *D* = 1.0, *D*_0_ = 0.01 and periodical boundary condition is used.

The results in Fig. 3 confirm that spiral wave still emerges in the network under noise in the case of periodical boundary condition, breakup of target wave, spiral wave can still be observed with increasing the intensity of noise, and disordered state emerges in the network when the intensity of noise is beyond certain threshold. Clearly, the spiral wave seems perfect as shown in Fig. 3(b) but never covers the network completely as the emergence spiral wave in reaction-diffusion systems, and it is important to check its stability by monitoring its development under different time units, and the results are shown in Fig. 4.

The results in Fig. 4 show that the spiral wave becomes stable with a transient period about 4000 time units, and it grows up quickly to occupy most of the areas in the network. That is to say, the formation of spiral wave induced by noise and autapses in local area could be much independent of the selection of boundary condition. Extensive numerical results show that noise plays an important role in growing up the spiral wave and thus bigger area of the network could be occupied by the spiral wave. The spiral wave shown in Fig. 4 is not as pefect as possible like the well-known spiral wave in reaction-diffusion system that spiral wave can cover the media completely. In fact, local distribution of electric autapse just induces spiral wave or segment in a local area but the spiral segment never grows up to occupy the network completely in the absence of noise, sometimes, broken spiral segments also emerges. Extensive numerical results seem to confirm that a developed spiral wave can cover more areas of the network by selecting noise intensity *D*_0_ = 0.01, which can be regarded as a more optimized noise intensity to develop a spiral wave and thus more neuronal activities can be regulated by the spiral wave. It is also important to investigate the effect of coupling intensity on the development of spiral wave at *D*_0_ = 0.01.

### Subsection B: Coupling intensity on the formation of spiral wave

**Figure 5:**
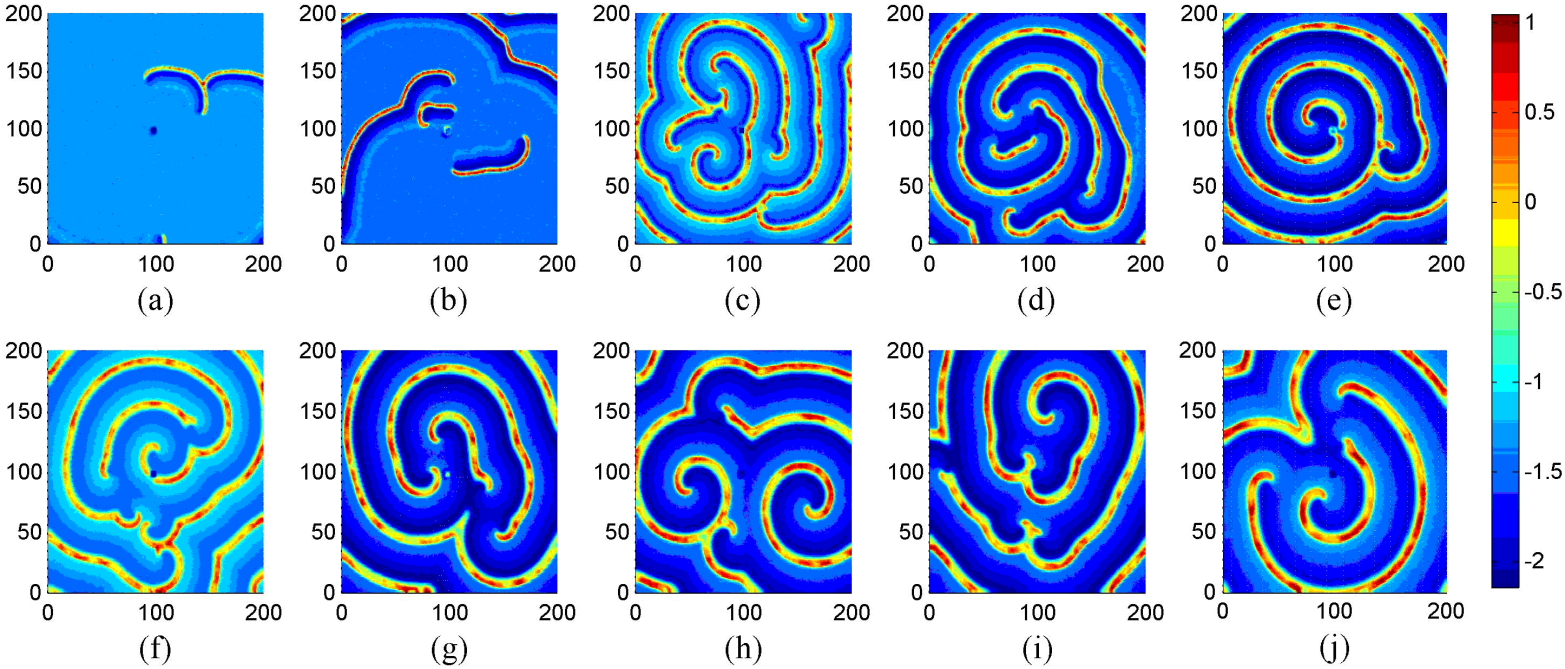
The developed states at *t* = 16000 time units in the network of neurons under different coupling intensities. For coupling intensity (a) *D* = 0.4, (b) *D* = 0.5, (c) *D* = 0.6, (d) *D* = 0.7, (e) *D* = 0.8, (*f*) *D* = 0.9, (*g*) *D* = 1.1, (h) *D* = 1.2, (i) *D* = 1.3, (*j*) *D* = 1.5. Where *g* = −1.5, *τ* = 30, *I_ext_* = 1.0, *D*_0_ = 0.01 and no-flux boundary condition is used.

Spiral wave is self-sustained, the propagation of spiral wave is much dependent on the coupling intensity, appropriate coupling intensity is much helpful for those spiral seeds, thus stable spiral wave can be developed more quickly from the spiral seed (or segment) under appropriate noise, and then more area of the network can be occupied by the spiral wave completely. For simplicity, it selectes the noise intensity *D*_0_ = 0.01, *I_ext_* = 1.0, *g* = −1.5, *τ* = 30, different coupling intensities are used to detect the development of spiral wave with a transient period about 16000 time units, and the results are shown in Fig. 5 when no-flux boundary condition is used for the network.

**Figure 6:**
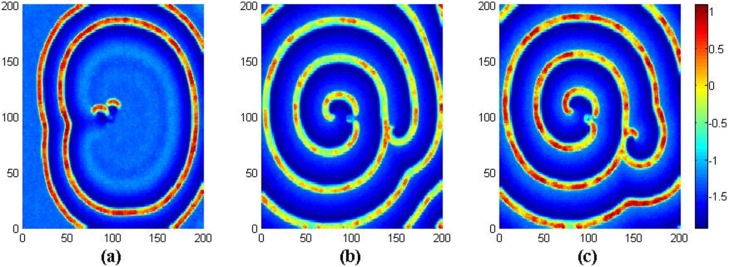
The development of spiral wave in the network. For (a) *t* = 2000, (b) *t* = 4000, (c) *t* = 16000 time units. Where *g* = −1.5, *τ* = 30, *I_ext_* = 1.0, *D* = 0.8, *D*_0_ = 0.01 and no-flux boundary condition is used.

The results in Fig. 5 show that no spiral wave can be developed in the network under smaller intensity of coupling, and spiral wave begins to emerge with increasing the coupling intensity in the case of no-flux boundary condition. To check the stability of spiral wave in Fig. 5(e), its development is monitored by illustrating the snapshots at different transient periods, and the results are shown in Fig. 6.

**Figure 7:**
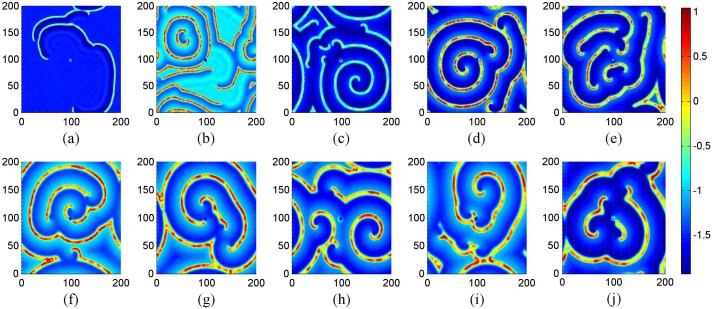
The developed states at *t* = 16000 time units in the network of neurons under different coupling intensities. For coupling intensity (a) *D* = 0.4, (b) *D* = 0.5, (c) *D* = 0.6, (d) *D* = 0.7, (e) *D* = 0.8, (*f*) *D* = 0.9, (*g*) *D* = 1.1, (h) *D* = 1.2, (i) *D* = 1.3, (*j*) *D* = 1.5. Where *g* = −1.5, *τ* = 30, *I_ext_* = 1.0, *D*_0_ = 0.01 and periodical boundary condition is used.

The results in Fig. 6 confirm that a spiral wave begins to emerge, and it occupies more and more nodes (size) vs. time, then it dominates most of the area in the network. It indicates that a perfect spiral wave induced by electric autapses in the network is also dependent on the selection of coupling intensity as the noise. It is also interesting to investigate this case under periodical boundary condition, and the results are shown in Fig. 7.

**Figure 8:**
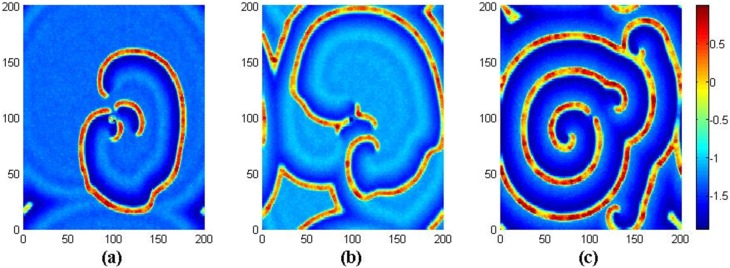
The development of spiral wave in the network. For (a) *t* = 2000, (b) *t* = 4000, (c) *t* = 16000 time units. Where *g* = −1.5, *τ* = 30, *I_ext_* = 1.0, *D* = 0.7, *D*_0_ = 0.01 and periodical boundary condition is used.

The results in Fig. 7 still show that spiral wave seldom emerges under smaller coupling intensity, and spiral wave begins to occur by using a stronger intensity of coupling. Then some perfect spiral wave is developed to occupy the network as shown in Fig. 7(d). However, more segments of spiral wave emerge in the network with increasing the intensity of coupling, and it shows some difference from the case of no-flux boundary condition. That is to say, perfect spiral wave emerges at *D* = 0.8 for no-flux boundary condition, while it emerges at *D* = 0.7 for periodical boundary condition. The potential mechanism could be that noise enhances the boundary condition effect. To detect the stability of the developed spiral wave in Fig. 7(d), snapshots under different transient periods are illustrated in Fig. 8.

The results in Fig. 8 show that periodical boundary condition is also effective to support stable spiral wave, and the spiral wave occupies most of the area in the network with a transient period about 6000 time units. It is also confirmed that spiral wave emerges well by selecting appropriate coupling intensity at fixed intensity of noise, gain, time delay in the autapse.

**Figure 9:**
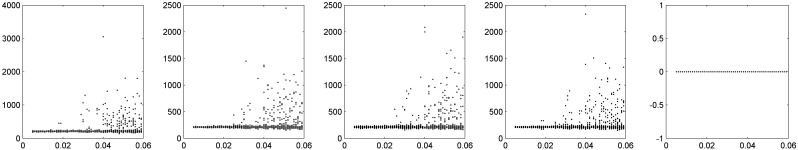
Bifurcation diagram for ISI vs. noise intensity. For (a) time series of membrane potentials in node (*i* = 90, *j* = 100), (b) time series of membrane potentials in node (*i* = 110, *j* = 100), (c) time series of membrane potentials in node (*i* = 100, *j* = 90), (d) time series of membrane potentials in node (*i* = 100, *j* = 110). No-flux boundary condition is used, and *D* = 1, *I_ext_* = 1.0, *τ* = 30, *g* = −1.5.

**Figure 10:**
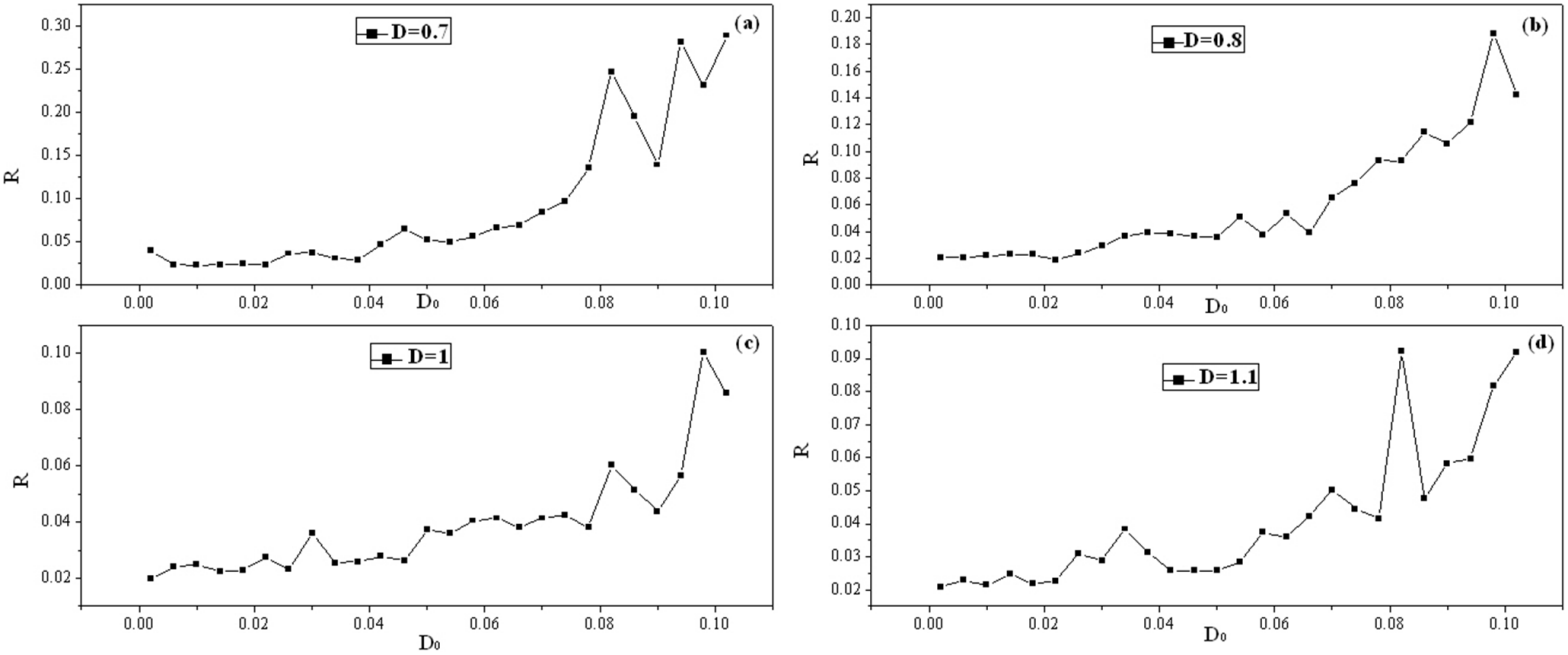
Distribution of factors of synchronization *vs*. noise intensity under no-flux boundary condition. For coupling intensity (a) *D* = 0.7, (b) *D* = 0.8, (c) *D* = 1, (d) *D* = 1.1. *I_ext_* = 1.0, *τ* = 30, *g* = −1.5, transient period t = 16000 time units, and the autapses are connected to neurons in the nodes (96≤*i, j*≤100).

Above all, the emergence of spiral wave in the network is just investigated by illustrating the snapshots under different autapse parameters (*g*, *τ*), coupling intensities and noise intensities. It is important to discern some statistical properties of the collective behaviors in the network by calculating the factors of synchronization. For simplicity, it will discuss the case for *D* = 1, *I_ext_* = 1.0, *τ* = 30, *g* = −1.5. The time series for four space symmetrical nodes (*i* = 90, *j* = 100), (*i* = 110, *j* = 100), (*i* = 100, *j* = 90), (*i* = 100, *j* = 110) are used for bifurcation analysis from the ISI (Inter-Spike Interval) *vs*. noise intensity D_0_∼[0.005∼0.1]. The bifurcation diagrams for the sampled time series vs., noise intensity are plotted in Fig. 9, and the distribution of factor of synchronization is plotted in Fig. 10.

The results in Fig. 9 show that the ISI values are much close to stable value about 250 when noise intensity is less than *D*_0_ = 0.03, thus the network can generate periodic or quasi-periodic property, which is necessary to support a stable spiral wave with distinct periodicity that can be verified by using time series analysis. The time series often shows distinct periodicity when spiral wave regulates or dominates the network, while disordered state emerges when breakup of spiral wave occurs under noise. However, the ISI begins to fluctuate in random that means no dominant period can be sustained in the network with further increasing the intensity of noise, as a result, no spiral wave can be developed and disordered states emerge in the network of neurons. To discern the phase transition of spiral wave in the network with no-flux boundary condition being used, the distribution for factors of synchronization is calculated by changing the intensity of noise carefully, and the results are shown in Fig. 10.

The results in Fig. 10 confirm that smaller factor of synchronization could be detected when the intensity of noise is low usually, and thus an ordered state could be reached, which is helpful to support a spiral wave in the network. The factor of synchronization tends to increase when stronger noise is used on the network, thus breakup of ordered wave occurs. Particularly, for the case *D*_0_ = 0.01, the factor of synchronization is approached by a much smaller value for *D* = 0.8 than *D* = 0.7, 1.0, 1.1 as well, which means a perfect spiral wave is much easy to be induced in the network. In the case for *D* = 0.7, the factor of synchronization fluctuates in the range from 0.02 to 0.04 when noise intensity is selected from *D*_0_ = 0.01 to 0.04. In the case for *D* = 0.8, 1.0, 1.1, slight deviation emerges for the factor of synchronization when noise intensity is changed below *D*_0_ = 0.06. In fact, spiral segments emerge in a local area of the network when smaller factors of synchronization are approached within [0.02, 0.04] though different area sizes can be covered by the spiral wave under different noise intensities. Compared the results in Fig. 10(a) with Fig. 10(b, c, d), it is found that the maximal factor of synchronization is decreased from 0.3→0.2→0.1 when the intensity is increased from *D* = 0.7→0.8→1.0→1.1 which makes the spiral segment propagate more quickly and regulate the collective behaviors of network more effectively. Extensive numerical results confirm that *D*_0_ = 0.01 can be the most effective noise intensity to enhance the growth of spiral wave so that more large number of neurons can be regulated by the spiral wave. By further increasing the noise intensity, it finds distinct shift in the curve for factor of synchronization, which means that the previous ordered state is removed and the network becomes disorder due to breakup of spiral waves. The case for periodical boundary condition is also investigated, and similar distribution of factor of synchronization is observed, that means smaller factor of synchronization vs. noise is active to support stable spiral wave in the network.

In a summary, autapses connected to neuron in a local area of the network are helpful to induce target wave, spiral wave emerges resulting from the breakup and collision of target waves, and optimized noise, coupling intensity are much important in supporting a stable spiral wave in the network. It accounts for the emergence of spiral wave in the neuronal network with autapse being considered. Time delay in autapse of a single neuron used to record the previous information, and the network enhances the memory and information propagation. The spiral wave is often self-sustained and robust to certain noise, thus the formation of spiral wave induced by autapse under optimized noise is much helpful for information memory in the network of neurons. It plays like a pacemaker and thus the collective electric behaviors of neurons are regulated by the spiral wave, which can be induced by appropriate distribution of electric autapses in the network.

## Conclusions

Autapses are observed in rat hippocampal neuron, and its functional roles are much attractive for study. The effect of autapse is often described by imposing self-feedback current with time delay on the membrane potential, and it is thought to be associated with memory and self-adjusting. In realistic neuronal system, a fraction of autapses can regulate the collective behaviors of neurons; therefore, it is interesting to study the transition and self-organization in neuronal network by detecting pattern dynamics. In this paper, the selection of spiral wave induced by autapses in a neuronal network is investigated, and the effect of noise on the network is also discussed. A statistical factor of synchronization is used to detect the transition of pattern induced by noise on the network, it is found that the emergence of spiral wave is often associated with a smaller factor of synchronization, which often represents an ordered state. Autapses connected to neurons in a local area can generate target wave in the network, breakup of target wave occurs due to noise, and optimized noise can enhance the ordered state by developing the broken target waves into a spiral wave, which regulates the collective electric behaviors of neurons like a pacemaker due to its intrinsic self-sustained dynamical property. Similar results are approached under no-flux and periodical boundary conditions.

## Supporting Information

Movie S1 **Supporting flash for spiral wave induced by autapses under periodical boundary condition.** (SWF)**.**
(GIF)

Movie S2 **Supporting flash for spiral wave induced by autapses under no-flux boundary condition.** (SWF) The two short movies are supplied to observe the formation of autapse-induced spiral waves in the network under different boundary conditions. Coupling intensity *D* = 1, noise intensity *D*_0_ = 0.01, *g* = −1.5, *τ* = 30, transient period *t* = 16000 time units.
(GIF)
