# Supplementary figures and images for: Autapse-Induced Spiral Wave in Network of Neurons under Noise
Source: PLoS One. 2014 Jun 26;9(6):e100849. doi: 10.1371/journal.pone.0100849 (PMC4072706; doi:10.1371/journal.pone.0100849)

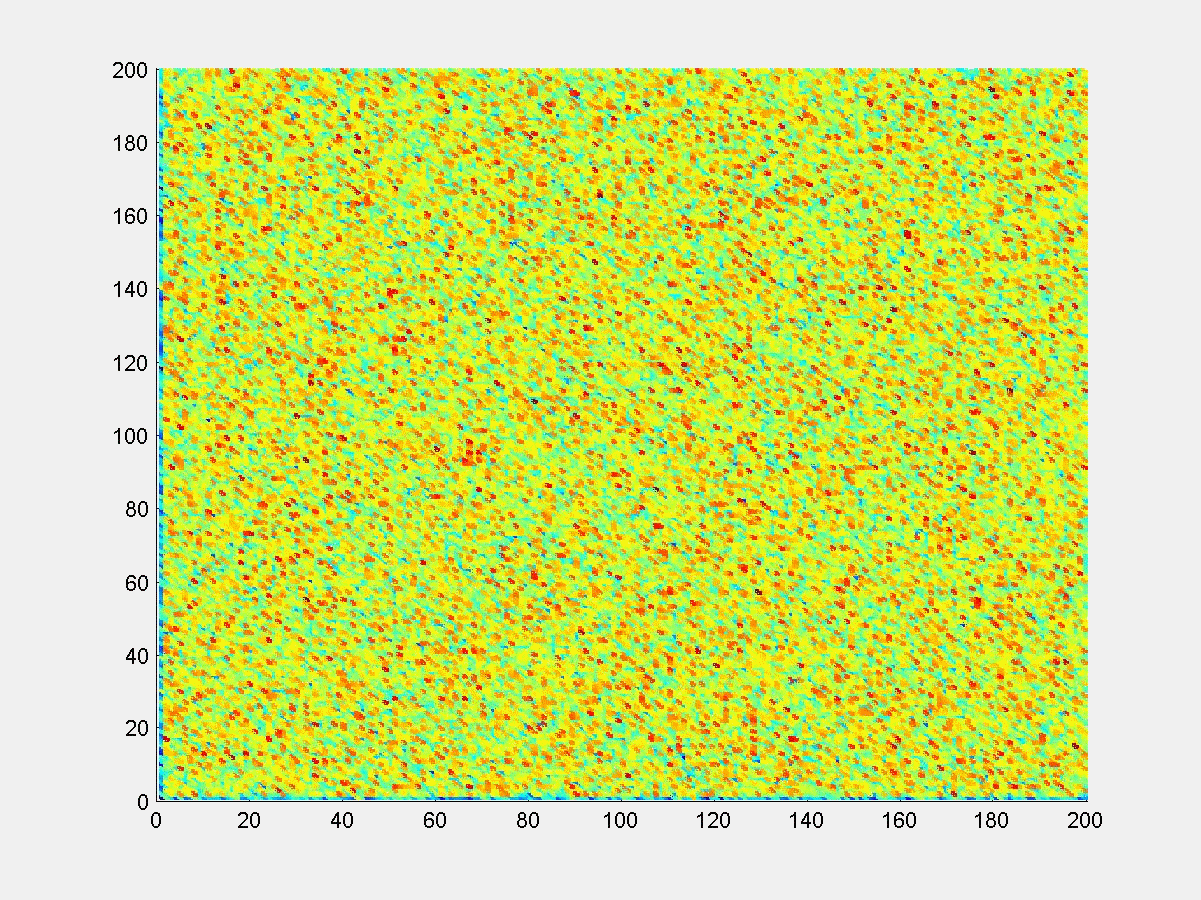

Supplement: Movie S1 — Supporting flash for spiral wave induced by autapses under periodical boundary condition. (SWF). (GIF) [file pone.0100849.s001.gif]

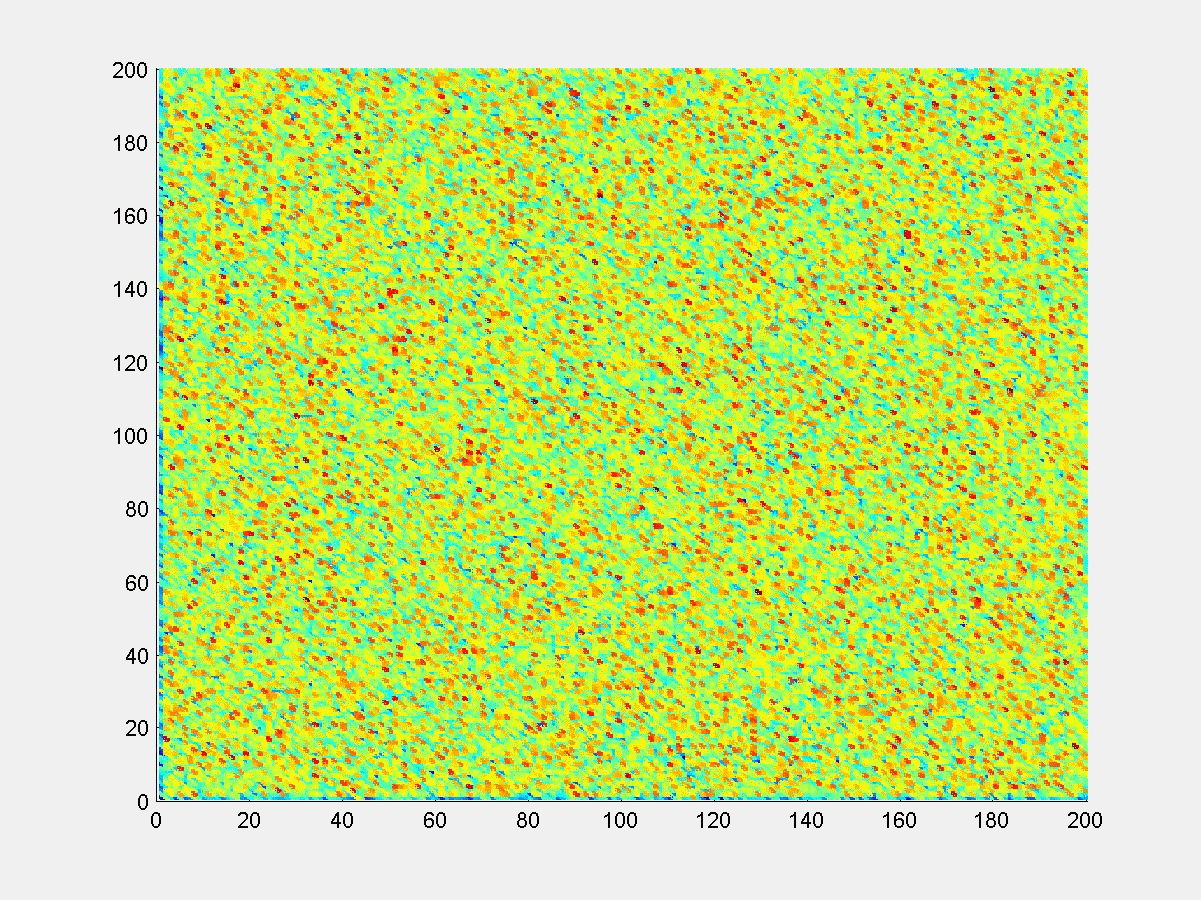

Supplement: Movie S2 — Supporting flash for spiral wave induced by autapses under no-flux boundary condition. (SWF) The two short movies are supplied to observe the formation of autapse-induced spiral waves in the network under different boundary conditions. Coupling intensity D = 1, noise intensity D 0 = 0.01, g = −1.5, τ = 30, transient period t = 16000 time units. (GIF) [file pone.0100849.s002.gif]
